# Comparison of Hay's Criteria with Nugent's Scoring System for Diagnosis of Bacterial Vaginosis

**DOI:** 10.1155/2013/365194

**Published:** 2013-06-13

**Authors:** Rohit Chawla, Preena Bhalla, Sanjim Chadha, Sujatha Grover, Suneela Garg

**Affiliations:** ^1^Centre of AIDS and Related Diseases, National Centre for Disease Control, 22 Shamnath Marg, New Delhi 110054, India; ^2^Department of Microbiology, Maulana Azad Medical College, Delhi University, Bahadur Shah Zafar Marg, New Delhi 110002, India; ^3^Department of Community Medicine, Maulana Azad Medical College, Delhi University, Bahadur Shah Zafar Marg, New Delhi 110002, India

## Abstract

Although Nugent's criterion is considered as the gold standard for the diagnosis of bacterial vaginosis (BV), the method requires an experienced slide reader and considerable time and skill. In this study, we compared the method of Hay and Ison with Nugent's scoring criteria. Vaginal specimens were collected from a total of 213 women, presenting with or without the symptoms of vaginitis. Diagnosis of BV was done using Nugent' and Hay's method. Sensitivity, specificity, and predictive values for positive and negative test were calculated for Hay's method using Nugent's method as the gold standard. We diagnosed 70 cases (32.86%) of BV by Nugent's method and 87 (40.85%) cases by the Hay's method. Sensitivity, specificity, predictive value of positive result, predictive value of negative result, and Kappa value when evaluating Hay's criteria using Nugent's criteria as the gold standard were ≥97.2%, ≥88.1%, ≥80.4%, ≥97.1%, and ≥0.830, respectively, when Hay's grade II and/or Nugent's intermediate score were considered either as negative or positive or excluded. Using Nugent score for the intermediate group is the most difficult. Hay's method shows good agreement with the gold standard method of Nugent et al. and can be used as an alternative to Nugent's criteria in busy tertiary care hospitals.

## 1. Introduction

Vaginal discharge is a common and distressful condition for a woman, which can result from a variety of physiological as well as pathological states [1]. The world over, bacterial vaginosis (BV) has been reported to be the most common cause of abnormal vaginal discharge among women in the reproductive age group [2–4]. BV is characterized by a change in the complex vaginal ecology, wherein the *Lactobacillus* dominant normal flora of the vagina is replaced by a mixed microbial flora consisting of anaerobes and *Gardnerella vaginalis *[5–7]. It is imperative to diagnose BV especially in pregnant females and institute treatment as early as possible to prevent complications such as low birth weight infants, preterm births [8], pelvic inflammatory disease [9], postpartum endometritis [10], and infertility [11]. Several epidemiologic studies indicate that BV is associated with increased susceptibility to HIV infection [12] and also several other sexually transmitted infections (STIs), including herpes simplex virus, gonorrhea, trichomoniasis, and chlamydia trachomatis infection [13–15]. 

A variety of methods were used for diagnosis until consensus was reached to define the diagnosis of BV using the composite criteria described by Amsel et al. [16]. An alternative method of diagnosis that has been used extensively is the grading or scoring of the microbial flora in the Gram-stained smears of vaginal fluid first described by Spiegel et al. in 1983 and later modified by Nugent et al. [17]. Both of these methods score the smears by quantification of the different vaginal morphotypes, making the evaluation of smears very subjective that requires an experienced slide reader and also considerable time and skill. Therefore, a simpler version was described by Ison and Hay in 2002 [18], in which vaginal flora is divided into the following three different categories: normal, intermediate, and BV depending on the relative amount of *Lactobacillus* morphotypes as compared to the *Gardnerella* morphotypes (Table 1).

We have been using the method of Nugent et al. for the last 15 years but have had to spend considerable time in training laboratory personnel before they could independently and accurately read the smears. The aim of the study was to evaluate the simple reading scheme of Hay et al. against the scoring method of Nugent et al. and to assess the utility of the former in terms of ease of training laboratory staff (laboratory technician and senior residents) and subsequently their ability in arriving at a correct diagnosis.

## 2. Materials and Methods

Patients included in the study were a part of a community-based study on prevalence of sexually transmitted infections/reproductive tract infections, carried out in two urban slums, one urban middle class colony and one rural area. The study had the approval from the ethics committee of Maulana Azad Medical College, New Delhi. Vaginal specimens were collected after obtaining informed consent from a total of 213 women aged between 15 and 49 years, presenting with or without the symptoms of vaginitis. Women who were menstruating, pregnant, or had received antibiotics in the past four weeks were excluded from the study.

Smears prepared from vaginal fluid were air-dried, heat-fixed, and Gram-stained. Briefly, the fixed smear was covered with crystal violet for 1 minute, washed with water, flooded with Gram's iodine for 1 minute, washed with water, and then decolorized with acetone for 2-3 seconds. The smears were rinsed quickly under running water to stop the decolorisation and then counterstained with safranin for 1 minute. The smear was rinsed with running water and blot-dried. All smears were assessed as per the grading systems of Nugent et al. and Hay et al. 

For diagnosis of bacterial vaginosis, Gram-stained vaginal smear was examined under oil immersion objective (1000x magnification) and graded as per standardized, quantitative, morphological classification developed by Nugent. Composite score was categorized into three categories, scores 0–3 being normal, 4–6 being intermediate, and 7–10 being definite bacterial vaginosis. For diagnosis of bacterial vaginosis by Hay's method, Gram-stained vaginal smear was examined under oil immersion objective (1000x magnification) and graded in the following manner: grade I (normal flora), *Lactobacillus* morphotype only; grade II (intermediate flora), reduced *Lactobacillus* morphotype with mixed bacterial morphotypes; grade III (bacterial vaginosis), mixed bacterial morphotypes with few or absent *Lactobacillus* morphotypes. In addition, the presence of clue cells was recorded.

Two hundred and thirteen vaginal smears were prepared from the same number of women. A highly experienced medical microbiologist (PB) initially examined all the smears which were later examined by a medical microbiologist with three years experience (RC) and a technical assistant with no experience in examining slides (SG). Inter-observer agreement was calculated between PB and RC and PB and SG.

*Statistical Analysis*. Sensitivity, specificity, and predictive values for positive and negative tests were calculated for Hay's method using Nugent's method as the gold standard. The measure of agreement was determined by Kappa index, where value of 1.0 indicates complete agreement. *P* values were determined using chi-square test and Fisher's exact test, as applicable.

## 3. Results

The results were analyzed by comparing both of the methods on the basis of observations recorded by PB. On the basis of Nugent's scoring, BV was diagnosed in 70 (32.9%) women, while 107 (50.2%) women had normal vaginal flora and 36 had an intermediate (mixed) flora (16.9%). 

Table 2 shows the comparison between Nugent's scoring system and Hay's classification system for the diagnosis of BV. All smears that had a score of ≥7 (BV) by Nugent's method were found to be of grade III (BV) by Hay's method. However, among the smears that were found to have a score of 4–6 (intermediate/mixed flora) by Nugent's method, 17 (47.2%) were placed in grade III (BV) and 3 (8.3%) were placed in grade I (normal) by Hay's method. Among those that were found to be normal by Nugent's method, 7 (6.5%) had grade II by Hay's method.

Discrepant results were obtained in 27 (12.7%) cases only and these were restricted only to nonbacterial vaginosis cases. Among these, seven smears which had discrepant results in the normal group had a score of 3. Out of twenty smears, which had discrepant result in the intermediate group, 9 had a score of 6, 5, had a score of 5 and 6 had a score of 4.

Sensitivity, specificity, predictive value of positive result, predictive value of negative result, and Kappa value when evaluating Hay's criteria using Nugent's criteria as the gold standard was ≥97.2%, ≥88.1%, ≥80.4%, ≥97.1%, and ≥0.830, respectively, when Hay's grade II and/or Nugent's intermediate score were considered either as negative or positive or excluded (Table 3). 

Presence of clue cells was found to be significantly associated with Hay's grade III (*P* < 0.001) and Nugent's score of 7–10 (*P* < 0.001) (Table 4). 

A better interobserver agreement (Table 5) was observed when the Hay and Ison method was used rather than Nugent's method for the diagnosis of bacterial vaginosis.

## 4. Discussion

Bacterial vaginosis has a high and varied prevalence, depending on the surveyed population, varying from 4% in developed countries to 61% in the so-called third world, with a mean prevalence of 14% considering developed and developing regions [19]. At least 30% of women presenting with vaginal complaints remain without a diagnosis even after a comprehensive workup [20]. Accurate diagnosis of BV is somewhat challenging. In addition to scientific considerations, choosing a method for its diagnosis requires consideration of complexity, cost, and the frequency of un-interpretable specimens [1].

Currently, the Nugent scoring method is the most frequently used laboratory-based diagnostic tool for detecting BV, and it is considered as the gold standard although its inter- and intraobserver reliabilities have been questioned [21]. The field size of the microscope has a bearing on the results which is another issue of concern [22]. Moreover, the score intervals are very narrow, with a difference of only a few bacteria, and the real number of *Lactobacillus* morphotypes recognized may be influenced by the variability of the methods. The homogeneity and thickness of the sample may vary in different ways of spreading the sample on the glass slide [23]. Therefore, it is important that the basic standards of quality control are adhered to.

In Hay's classification system, a quantitative estimation of the bacterial morphotypes is not done; instead, an evaluation of the relationship between the amounts of bacteria is conducted. The field size of the microscope does not have an influence on the results [21]. Besides, this classification system can be used on slides stained with different staining methods and also on smears with no stains. But the robustness of this system must be assessed in different settings and populations around the world and when performed by different readers. To the best of our knowledge, this is the first study done to evaluate the simple reading scheme of Hay et al. against the scoring method of Nugent et al. in India.

In our study, there was a strong association of a normal Nugent's score with grade I flora and a Nugent's score of ≥7 with grade III Hay's classification, with only 6.5% of women with a Nugent's score of ≤3 falling in grade II by Hay's method. Controversy occurred in women with intermediate scores in Nugent's method (47.2% being placed in grade III and 8.3% in grade I by Hay's method). Therefore, more women were diagnosed with BV by the Hay's method. However, among the 70 women diagnosed BV by Nugent's method, 70% were clue cell positive while among 87 women who were grade III by Hay's method, only 57.5% women were clue cell positive. We also found high sensitivity, specificity, predictive values, and Kappa indexes for Hay's criteria as compared to the reference method of Nugent et al. in our study indicating that the two methods are very alike. These results indicate that when there is a lack of time or expertise, this method of assessment of microbial flora can be used as an alternative method of diagnosis. Similar results have been reported by Larsson et al. from Sweden [22]. 

Our study also shows that clue cells were significantly associated with Hay's grade III (*P* < 0.001) and Nugent's score of 7–10 (*P* < 0.001). Another investigator from Kolkata, India, has reported that the presence of clue cells correlates best with a positive diagnosis by Nugent's score [1]. This result indicates that by adding a score for the presence/absence of clue cells to each of these 2 methods, one can enhance the diagnosis of BV, which can be superior to the methods of Nugent et al. and Hay et al.

## 5. Conclusions

The Nugent scoring system is reliable in diagnosing either BV negative or positive smears, but a problem arises in the intermediate group which is the most difficult to interpret. In a developing country with limited resources such as India, where highly trained skilled manpower comes at a premium, diagnosis of BV by Nugent's score would place a great strain on available resources. Therefore, there is a great need for an inexpensive diagnostic method that is reliable and unifies clinical and microbiological parameters to make it more sensitive while retaining its specificity.

Hay's classification system seems to constitute a good classification method, as it allows the microscopist to formulate an impression based on the relative numbers of *Lactobacilli* and *Gardnerella* morphotypes so that the influences of surface area and bacterial density are lessened. This simplified assessment of Gram-stained smears can be used as an alternative to Nugent's scoring and is more applicable for use in busy tertiary care hospitals catering to large populations, as this method has demonstrated good agreement with the gold standard method of Nugent et al.

## Figures and Tables

**Table 1:** The Hay/Ison classification.

	*Lactobacilli* morphotypes	*Gardnerella* morphotypes
Normal (group 1)	Many	Few
Intermediate (group 2)	Equal amount	Equal amount
BV (group 3)	Few	Many

**Table 2:** Comparison of Hay's classification system with Nugent's scoring system for diagnosis of BV in study subjects (*n* = 213).

Hay's grading	Nugent's score			
	Normal (0–3)	Intermediate (4–6)	Positive (≥7)	Total number (%)
I	100	3*	0	103 (48.4)
II	7**	16	0	23 (10.8)
III	0	17***	70	87 (40.8)

Total number (%)	107 (50.2)	36 (16.9)	70 (32.9)	213

*Two had Nugent's score of 4, and 1 had Nugent's score of 5.

**All 7 had Nugent's score of 3.

***Nine had Nugent's score of 6, four had Nugent's score of 5, and four had Nugent's score of 4.

**Table 3:** Sensitivity, specificity, and predictive values of Hay's classification system using Nugent's score as the gold standard for the diagnosis of BV in study subjects.

	1	2
Sensitivity (%)	100	97.2
Specificity (%)	88.1	93.4
Predictive value of positive result (%)	80.4	93.7
Predictive value of negative result (%)	100	97.1
Kappa	0.830	0.906

(1) Hay's: grade I = negative; grade II = negative; grade III = positive, Nugent's: score 0–3 = negative; score 4–6 = negative; score 7–10 = positive.

(2) Hay's: grade I = negative; grade II = positive; grade III = positive, Nugent's: score 0–3 = negative; score 4–6 = positive; score 7–10 = positive.

**Table 4:** Presence of clue cells in relation to Nugent's scoring system and Hay's classification system.

	*n*	Clue cells		*P* value Odds ratio
		Positive	Negative	
Nugent's score				
7–10	70	49	21	<0.001
4–6	36	2	34	164.5
0–3	107	0	107	

Hay's score				
III	87	50	37	<0.001
II	23	1	22	168.9
I	103	0	103	

**Table 5:** Interobserver agreement (Kappa value)*.

Inter-observer agreement	Nugent's method		BV by Hay's	
	BV	Normal	Grade I	Grade III
Between PB and RC	0.82	0.75	0.91	0.88
Between PB and SG	0.67	0.63	0.72	0.76

*Intermediate scores in Nugent's and grade II in Hay's and Ison's classification have been excluded for calculating interobserver agreement.
